# Structural and Mutational Studies on Substrate Specificity and Catalysis of *Salmonella typhimurium* D-Cysteine Desulfhydrase

**DOI:** 10.1371/journal.pone.0036267

**Published:** 2012-05-04

**Authors:** Sakshibeedu R. Bharath, Shveta Bisht, Rajesh K. Harijan, Handanahal S. Savithri, Mathur R. N. Murthy

**Affiliations:** 1 Molecular Biophysics Unit, Indian Institute of Science, Bangalore, India; 2 Department of Biochemistry, Indian Institute of Science, Bangalore, India; National Institute for Medical Research, Medical Research Council, London, United Kingdom

## Abstract

*Salmonella typhimurium* DCyD (*St*DCyD) is a fold type II pyridoxal 5′ phosphate (PLP)-dependent enzyme that catalyzes the degradation of D-Cys to H_2_S and pyruvate. It also efficiently degrades β-chloro-D-alanine (βCDA). D-Ser is a poor substrate while the enzyme is inactive with respect to L-Ser and 1-amino-1-carboxy cyclopropane (ACC). Here, we report the X-ray crystal structures of *St*DCyD and of crystals obtained in the presence of D-Cys, βCDA, ACC, D-Ser, L-Ser, D-cycloserine (DCS) and L-cycloserine (LCS) at resolutions ranging from 1.7 to 2.6 Å. The polypeptide fold of *St*DCyD consisting of a small domain (residues 48–161) and a large domain (residues 1–47 and 162–328) resembles other fold type II PLP dependent enzymes. The structures obtained in the presence of D-Cys and βCDA show the product, pyruvate, bound at a site 4.0–6.0 Å away from the active site. ACC forms an external aldimine complex while D- and L-Ser bind non-covalently suggesting that the reaction with these ligands is arrested at Cα proton abstraction and transimination steps, respectively. In the active site of *St*DCyD cocrystallized with DCS or LCS, electron density for a pyridoxamine phosphate (PMP) was observed. Crystals soaked in cocktail containing these ligands show density for PLP-cycloserine. Spectroscopic observations also suggest formation of PMP by the hydrolysis of cycloserines. Mutational studies suggest that Ser78 and Gln77 are key determinants of enzyme specificity and the phenolate of Tyr287 is responsible for Cα proton abstraction from D-Cys. Based on these studies, a probable mechanism for the degradation of D-Cys by *St*DCyD is proposed.

## Introduction

Pyridoxal 5′ phosphate (PLP)-dependent enzymes catalyze several reactions in the metabolism of amino acids. The reactions catalyzed include the transfer of amino group, decarboxylation, removal or replacement of chemical groups at α, β or γ positions, and inter-conversion of L and D forms of amino acids [1]. PLP-dependent enzymes have been classified into three groups (α, β, and γ families) based on the carbon atom at which the net reaction takes place [2]. They have also been classified into four structurally distinct folds (I–IV) [3]. The majority of PLP-dependent enzymes that catalyze elimination and replacement reactions with amino acids are specific to L-amino acids. However, there are some PLP-dependent enzymes (e.g. D-serine dehydratase from *Salmonella typhimurium* [4]) that catalyze such reactions with D-amino acid substrates. Although D-amino acids are not used in protein biosynthesis, they are nevertheless key components of several biomolecules such as antibiotics and bacterial cell wall. D-cysteine (D-Cys) moieties are found in firefly luciferin, semisynthetic cephalosporin MT141 and malformin A [5], [6]. D-cysteine desulfhydrase (DCyD) is suggested to take part in the catabolic degradation of D-Cys and D-Cys-containing toxic compounds [7].

The extensively studied amino acid dehydratases belong to the β family or fold type-II of PLP dependent enzymes [3]. These enzymes catalyze the irreversible deamination of amino acids to the respective α-keto acids. L-serine and L-threonine dehydratases have been purified and characterized both biochemically and structurally from several organisms [8], [9], [10]. Extensive structural and functional studies have been carried out on *Salmonella typhimurium* D-serine dehydratase [4]. Most of the amino acid dehydratases utilize PLP based chemistry for catalysis while there are a few deaminases which use iron-sulfur clusters instead of PLP for catalysis [11].

DCyD from *E. coli* (*Ec*DCyD) carries out degradation of D-Cys, β-chloro-D-alanine (βCDA) and β-substituted D-cysteine derivatives to the respective keto acids [7]. Except for a low level of activity with D-Ser, *Ec*DCyD does not carry out elimination reaction with other D- or L-amino acids or their substituted compounds [7]. *Ec*DCyD has also been reported to carry out β-replacement reaction with βCDA in the presence of high concentrations of different thiols to form D-Cys and other substituted D-Cys compounds [7]. Although detailed biochemical studies on *Ec*DCyD have been carried out, its three-dimensional structure has not yet been determined.

*Salmonella typhimurium* DCyD (*St*DCyD) shares 90% sequence identity with *Ec*DCyD. DCyD is homologous to 1-amino-1-carboxy cyclopropane (ACC) deaminase (ACCD), although their specificity and mechanistic features are not similar. Crystal structures of ACCD from *Hansenula saturnus* (*Hs*ACCD; PDB code 1F2D) [12] and *Psuedomonas spp.* (*Ps*ACCD; PDB code 1TYZ) [13] and a homolog of ACCD from *Pyrococcus horikoshii* (*Ph*AHP; PDB code 1J0A) [14] have been reported. *St*DCyD amino acid sequence is 33%, 36%, and 39% identical to those of *Hs*ACCD, *Ps*ACCD and *Ph*AHP, respectively.

In this manuscript, we describe X-ray crystal structures of *St*DCyD and its complexes with various ligands and present comparative analyses with other PLP dependent enzymes. The role of residues close to the ligand binding pocket in the catalytic function of *St*DCyD has been probed by mutational and functional studies. The results of these experiments are described.

## Results and Discussion

### Biochemical studies on *St*DCyD

Recombinant *St*DCyD was expressed in *E. coli* with a hexa-histidine tag and purified by Ni-NTA affinity and size exclusion chromatography. The purified protein was yellow in colour with an absorbance maximum at 417 nm, indicating that the enzyme contains PLP at the active site. The purified enzyme was tested for activity with D-Cys, βCDA, D-Ser, L-Ser, ACC and D-Ala. The enzyme effectively catalyzed the degradation of only two of these compounds (D-Cys and βCDA). With D-Cys, the K_m_ and V_max_ of the enzyme were 0.34±0.03 mM and 220.6±46.9 µmol pyruvate produced/min/mg of protein, respectively. Significant activity was also observed with βCDA (K_m_ = 0.84±0.30 mM, V_max_ = 173.4±82.1 µmol pyruvate produced/min/mg of protein). The homologous enzymes, *Hs*ACCD [15] and *Ps*ACCD [13] degrade ACC but not D-Cys or D-Ser. There is evidence that *Ps*ACCD degrades βCDA also [13]. *Ph*AHP has been shown to catalyze the degradation of both D-Ser and L-Ser to pyruvate. It has no activity with ACC or D-Cys [14]. Thus, these homologous enzymes display intriguing differences in substrate specificities and enzymatic functions.

**Figure 1:**
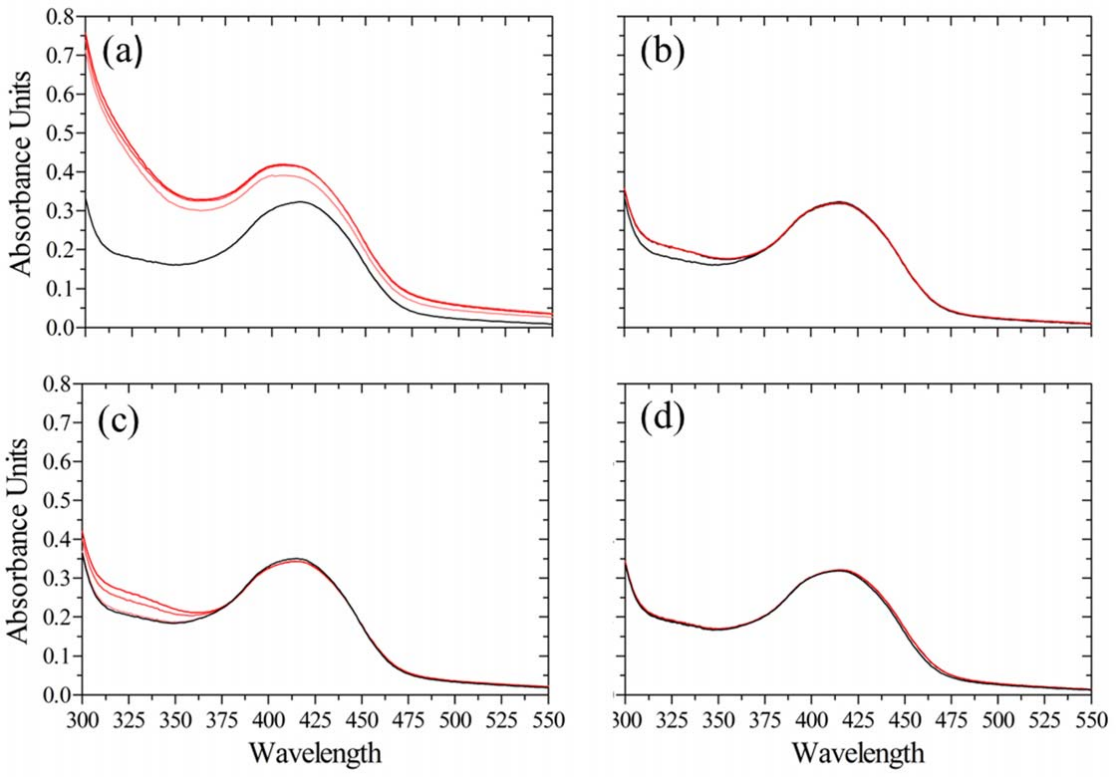
Spectroscopic changes observed after incubation of *St*DCyD with ligands. (a) D-Cys, (b) βCDA, (c) D-Ser and (d) L-Ser. Spectra before adding the substrate is shown in black. Red lines from bottom to top represent spectra at 1, 5 and 10 min after adding the substrate. Large changes are observed at ∼330 nm upon the addition of D-Cys. Changes observed with βCDA and D-Ser are similar but distinct from the changes observed with D-Cys. No spectral changes are observed with L-Ser.

Binding of D-Cys, βCDA, D-Ser, L-Ser and ACC to *St*DCyD were examined by monitoring spectral changes (300–550 nm) for 20 min upon addition of these ligands to the purified enzyme (Fig. 1). Large spectral changes were observed with D-Cys while changes observed with 10 mM βCDA or 40 mM D-Ser were marginal. In contrast, addition of 10 mM ACC or 40 mM L-Ser did not lead to spectral changes. The increase in the absorption at 330 nm observed with D-Cys corresponds to the formation of the product pyruvate.

### Quality of *St*DCyD structural models

Structures of two distinct crystal forms of recombinant *St*DCyD were determined by the molecular replacement method. In the crystal form I (PDB code: 4D8T), the asymmetric unit contained four protomers (A, B, C and D). Except for a few residues at the N terminus of B and C subunits, electron density was of good quality throughout the polypeptide chain in all the protomers. The structure of form II (PDB code: 4D8U) contained eight protomers in the asymmetric unit and was of considerably lower resolution (3.3 Å as opposed to 2.2 Å of form I). Therefore, Form II structure has been utilized only for qualitative analysis. Apart from the unliganded forms, structures of nine enzyme-ligand complexes have also been determined in crystal form I. In all the eleven structures of form I, besides the hexa-histidine tag, six residues at the N-terminus in chains B and C did not have well-defined electron density and hence have not been included in the final model. All the residues of A and D chains were in good electron density. In the form I native structure, 97.1% and 99.7% were in the most favored and allowed regions, respectively, of the Ramachandran plot [16]. Two residues were considered as outliers by MOLPROBITY [17]. These residues are Glu181 and Glu182 of chain D. Residues 181 and 182 are surface exposed glutamates with disordered side chains.

### Structure of *St*DCyD

**Figure 2:**
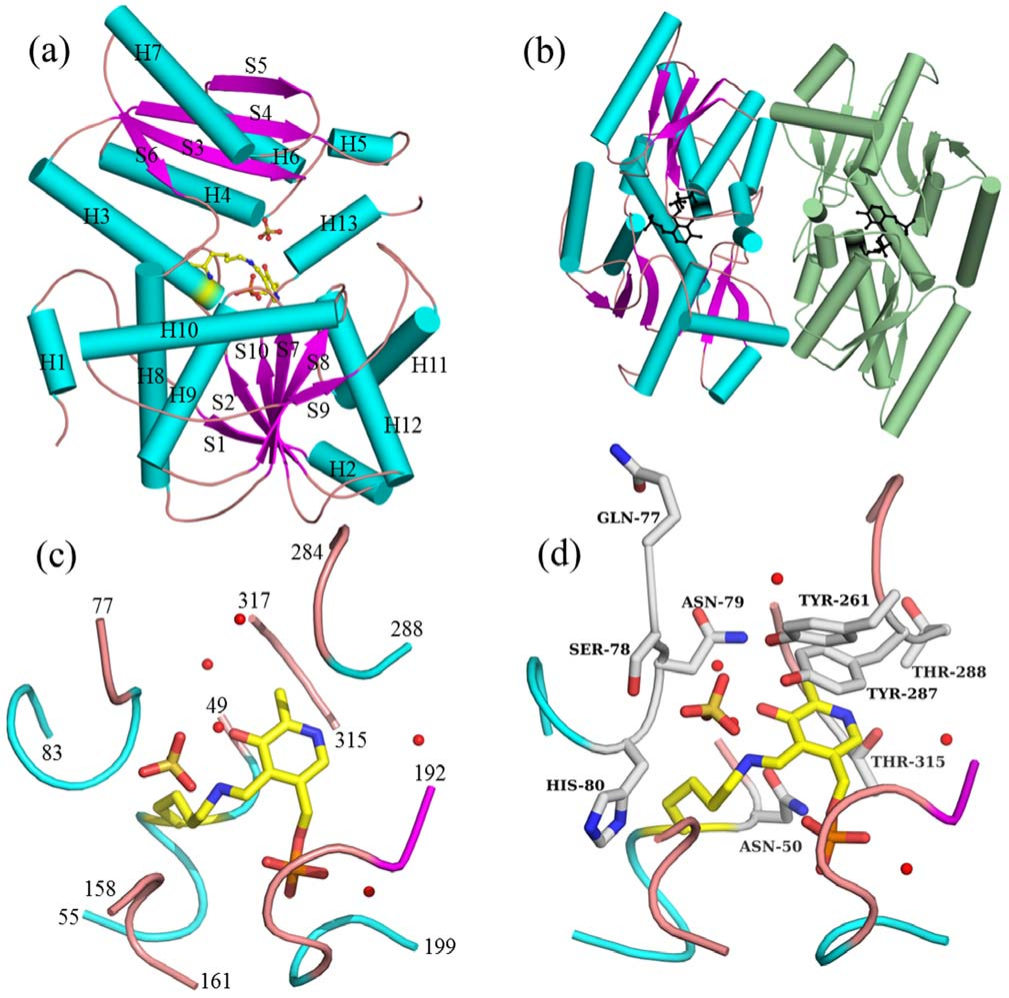
Structure of *St*DCyD. (a) Polypeptide fold of *St*DCyD illustrated as a ribbon diagram. α- helices are shown in cyan, β-strands in magenta and loops in brown. PLP at the active site is shown in ball and stick representation. (b) Dimeric structure of *St*DCyD. The two protomers are coloured differently. The enzyme forms a compact dimer similar to those of other fold type II PLP-dependent enzymes. Active sites of the two monomers are marked by PLP, shown in ball and stick representation. (c) Active site loops. Residues 49–55, 77–83, 158–161, 192–199, 284–288 and 315–317 are shown along with lysine linked PLP, sulphate ion and active site water molecules (spheres). (d) Geometry of the active site. Residues Asn50, Gln77, Ser78, Asn79, His80, Tyr261, Thr287, Thr288 and Thr315 are shown in ball and stick along with PLP linked to Lys51, sulphate ion and active site water molecules (spheres). The orientation is the same as that of (c).

The tertiary structure of *St*DCyD protomer is shown in Figure 2A. The secondary structural elements (as defined by the program DSSP [18]) are shown in cartoon representation. The polypeptide fold of *St*DCyD is similar to those of other fold type II PLP-dependent enzymes and consists of a small domain (residues 48–161) and a large domain (residues 1–47 and 162–328). The small domain folds as an open twisted α/β structure consisting of a central four-stranded (S3–S6) parallel β-sheet and five surrounding helices (H3–H7). The large domain contains seven α-helices (H1, H2, and H8–H12) and six β-strands (S1, S2, and S7–S10). In the central β-sheet of the large domain, all strands except for one short terminal strand (S1) are parallel. These strands are strongly twisted such that the sixth strand is at angle of 122° to the first. The C-terminal helix H13 protrudes away from this domain and interacts with the small domain. Except for β-strands at the extremities of the sheets, all strands are largely protected from the solvent by the surrounding helices, and all β-strands of the large domain point towards the molecular center. There are four β-α-β motifs, 18 β turns, two γ turns and one β hairpin in the polypeptide. The root mean square deviation (rmsd) of corresponding Cα atoms in the pair wise superposition of the polypeptide backbone of subunits present in the asymmetric unit in form I crystal structures vary between 0.20 to 0.60 Å. DALI search revealed that the tertiary structure of *St*DCyD is most similar to those of ACC deaminases *Ps*ACCD and *Hs*ACCD and to the ACC deaminase homolog *Ph*AHP. Structural superposition of Cα atoms of *St*DCyD with the corresponding atoms of *Ps*ACCD, *Hs*ACCD and *Ph*AHP polypeptides resulted in rmsds of 1.31, 1.44 and 1.47 Å, respectively.

Gel filtration studies suggest that *St*DCyD is a dimer in solution. In the form I structure, there are two dimers (AB and CD) and in form II structure, there are four dimers (AB, CD, EF and GH) in the asymmetric unit that are similar to the dimeric organization found in other fold type II PLP-dependent enzymes. The two protomers of *St*DCyD dimer interact closely at their nearly flat surfaces (Fig. 2B). Accessible surface area calculations show that a single subunit of *St*DCyD with bound PLP has a surface area ranging from 13,004 to 13,240 Å^2^. The total surface area buried on dimerization is 1518 Å^2^ (11.7%) and 1487 Å^2^ (11.3%) per protomer in AB and CD dimers, respectively. Analysis of the dimeric structure by PISA [19] revealed 42–46 residues from each protomer at the dimer interface, forming hydrophobic interactions and nine hydrogen bonds. The interface consists of a hydrophobic core formed by Met47, Leu282, Pro319, Leu321 and Phe322. This core is further surrounded by hydrophobic residues Leu117, Leu118, Leu121 and Phe122. In each subunit, residues from H2 (25–26), H4 (86, 89–92), H5 (109), H6 (120–123), H12 (272–273, 276–277, 279–280, 282), H14 (318–319, 321–322, 325–326), S1 (20, 22), loop between S1 and H3 (39, 42, 44–47) and loop between H5 and H6 (112–114, 117–118) contribute to the dimer formation. Hydrogen bonds, which are common to interfaces of both AB and CD dimers, are between Arg26–Ala89, Arg39–Pro44, Asn109–Asn109, Asn113–Phe322 and Lys273–Asn123.

### *St*DCyD Active site

As in other PLP-dependent fold type II enzymes, the active site is situated in a large crevice between the two domains (Fig. 2A). PLP is bound by a Schiff base linkage to Lys51. It is surrounded by segments consisting of residues 49–55, 77–83, 158–161, 192–199, 315–317, 287–288 and 284 (Fig. 2C). The pyridine ring of PLP is stacked by the aromatic ring of Tyr287 (Fig. 2D) on one side and interacts with the side-chain of Asn50 on the other side. Tyr287 hydroxyl is involved in hydrogen bonding with Tyr261 hydroxyl, which in turn is hydrogen bonded to a water molecule. These interactions could reduce the pKa of Tyr287 and enable it to be in its phenolate form. Thus, apart from stacking with the pyridine ring, Tyr287 may also have a role in the deprotonation of the incoming substrate as in *Hs*ACCD [15]. In several fold type II PLP-dependent enzymes, the residue immediately preceding PLP-bound Lys is an aromatic or a hydrophobic residue (His86 in TRPSβ [20]; Phe61 in TD [8] and Val40 in OASS [21]). In *St*DCyD, like in ACCDs, an asparagine (Asn50) residue precedes Lys51. The PLP phosphate is held by hydrogen bonding with main chain nitrogen atoms of residues Gly194, Ser195, Ala196, Gly197 and Thr198. A water molecule is present at hydrogen bonding distance (2.60 Å) to O3 of phosphate. This water and the glycine-rich loop are conserved in several other PLP-dependent enzymes. The pyridine nitrogen of PLP is within hydrogen bonding distance (2.54 Å) from the side-chain hydroxyl group of Thr315.

Electron density map of unliganded *St*DCyD revealed a significant patch of density with tetrahedral geometry at the active site that could be interpreted as a sulphate (in form I crystal structure) or a phosphate (in form II crystal structure). Form I crystals were obtained in a condition which had sulphate while conditions for obtaining form II had phosphate ions. These ions could be refined to reasonable B factors that were close to those of the surrounding atoms.

### Active site comparisons

**Figure 3:**
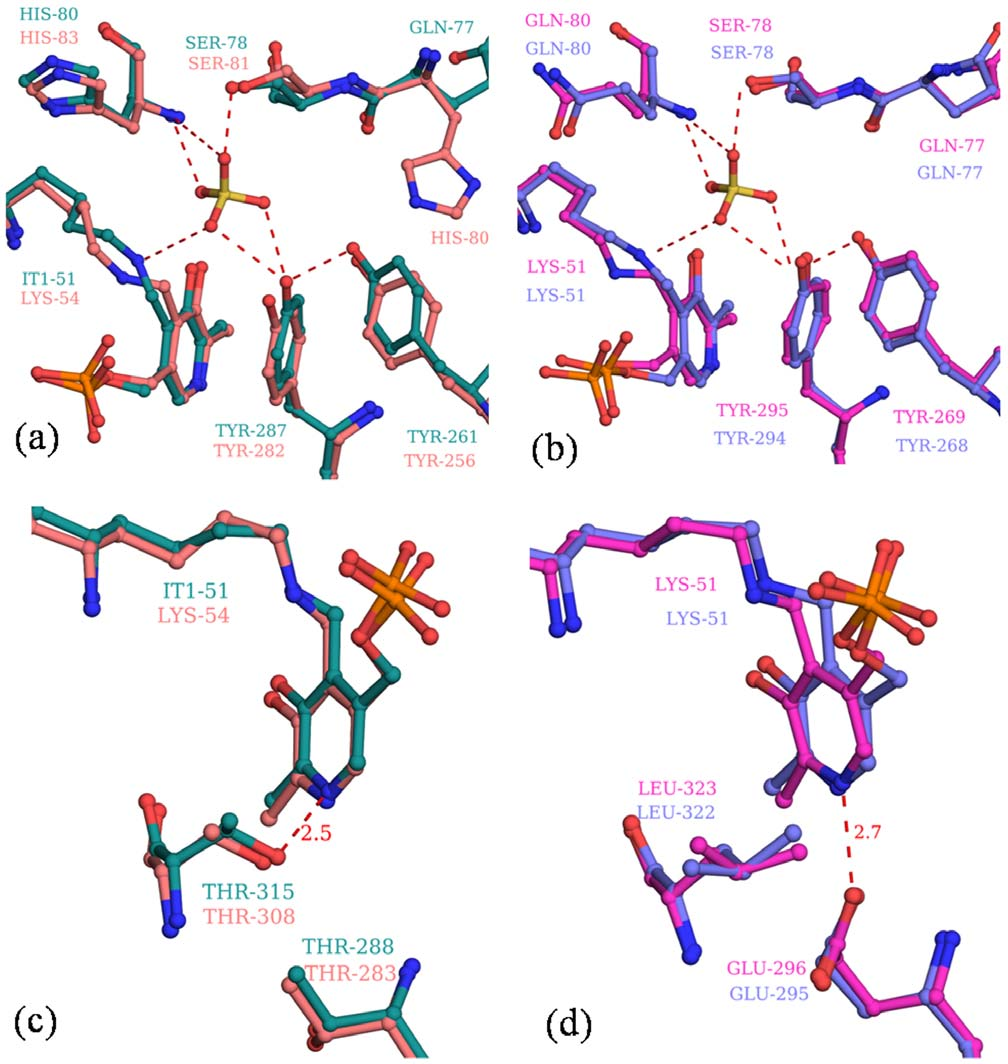
Structural differences at the active site of *St*DCyD and its homologs. Comparison of the active sites of (a) *St*DCyD (green) and *Ph*AHP (brown) (b) *Ps*ACCD (magenta) and *Hs*ACCD (blue). Hydrogen bonds involving sulphate oxygens are shown. Comparison of the chemical environment near PLP N1 of (c) *St*DCyD (green) and *Ph*AHP (brown) (d) *Ps*ACCD (magenta) and *Hs*ACCD (blue) is shown. Hydrogen bonding interaction of N1 is shown as a dashed line.

Superpositions of the active site residues of *St*DCyD and *Ph*AHP; and *Ps*ACCD and *Hs*ACCD are shown in Figure 3A and 3B, respectively. The lysine residue which forms the internal aldimine is conserved in all PLP dependent enzymes. His80 of *St*DCyD is conserved in *Ph*AHP (His83, Fig. 3A) and replaced by glutamine in *Ps*ACCD (Gln80) and *Hs*ACCD (Gln80, Fig. 3B). Gln77 of *St*DCyD is conserved in *Ps*ACCD (Gln77) and *Hs*ACCD (Gln77), but replaced by a histidine (His80) in *Ph*AHP. The side chain orientations (as defined by χ_1_) of the structurally equivalent residues Gln77 of *St*DCyD and His80 of *Ph*AHP are completely different. Side chain of His80 of PhAHP faces the substrate binding pocket and is hydrogen bonded to the hydroxyl group of Tyr256 (equivalent to Tyr261 of *St*DCyD), whereas in *St*DCyD, Gln77 faces away from the substrate binding pocket. If His80 of *Ph*AHP assumes the conformation found for Gln77 in *St*DCyD, the side chain of His80 would make short contacts with the side chain of His316. The short side chain of Ala323 (structurally equivalent to His316 of *Ph*AHP) in *St*DCyD is thus responsible for the observed conformation of Gln77. The importance of these observations is discussed in the context of mutagenesis studies in the section on D- and L-Ser complexes of *St*DCyD. The pyridine nitrogen atom of PLP in *St*DCyD is hydrogen bonded to the hydroxyl of Thr315 (Fig. 3C). A similar hydrogen bonding with Thr308 is observed in *Ph*AHP. This threonine is replaced by a leucine in *Ps*ACCD and *Hs*ACCD (Fig. 3D). In these structures, the pyridine nitrogen is hydrogen bonded to side chain carboxylates of Glu295 and Glu296, respectively (Fig. 3D). As in *St*DCyD and *Ph*AHP, in most other fold type II PLP-dependent enzymes also, the pyridine N is hydrogen bonded to a Thr or a Ser hydroxyl group. The nature of the group hydrogen bonding with pyridine N is likely to determine the electron withdrawing power of PLP [22]. Hydrogen bonding to a negatively charged group such as a carboxylate is likely to enhance the electron withdrawing power of PLP. This could stabilize a quinoniod intermediate in the course of catalysis. Thus, a quinonoid intermediate is detected in some of the fold type I enzymes but not usually in fold type II enzymes.

### Catalytic properties of mutants

Examination of the active site geometry and comparison with ACCDs suggested that residues Thr315, Thr288, Tyr287, Tyr261, His80, Gln77 and Ser78 that are close to the active site pocket may be important for catalysis. The residues equivalent to Tyr287 and Ser78 have been shown to be involved in the initial steps of catalysis in ACCDs [13], [15]. Thr315 and Thr288 may be important for substrate specificity [23]. Gln77 and His80 could be important for activity with D-Ser [14]. The roles of these residues were further probed by constructing single site mutants T288E, Y287F, Y261F, H80Q, Q77H, S78A and the double site mutant T315L/T288E.

**Figure 4:**
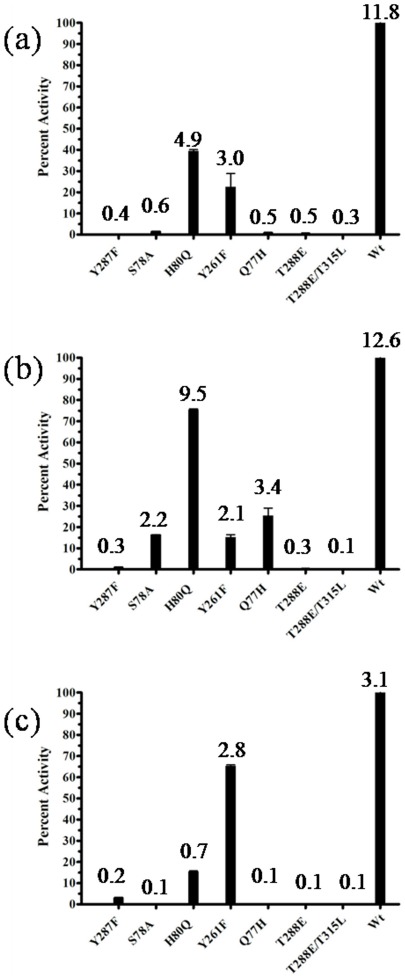
Catalytic properties of *St*DCyD. The bar diagram shows the residual catalytic activity (%) with respect to the wild type (100%) of the active site mutants of *St*DCyD towards (a) 2 mM D-Cys, (b) 5 mM βCDA, and (c) 50 mM D-Ser. Specific activity values (expressed as µmoles of pyruvate formed per min per mg of protein) are given above the bar graphs. The observed activities suggest that Gln77 and Ser78 contribute to specificity with respect to D-Cys and βCDA.

The enzymatic activities of the mutants are shown in Figure 4. Y287F was inactive with respect to all substrates suggesting that Tyr287 is a crucial residue for the function of *St*DCyD. Karthikeyan et al. [13], have shown that the equivalent residue in *Ps*ACCD (Tyr294) carries out the deprotonation step of catalysis with βCDA. Thus, it is reasonable to assume that Tyr287 of *St*DCyD is also involved in the deprotonation of Cα of the substrate external aldimines of D-Cys, βCDA and D-Ser. The mutants S78A and Q77H were inactive with respect to D-Cys, but retained significant activity with βCDA (S78A retained 20% activity while Q77H retained 30% activity) indicating that Ser78 and Gln77 are key residues in distinguishing the two substrates. The loss of activity (75–85%) with respect to D-Cys and βCDA were comparable in Y261F mutant while it retained substantial activity (70%) with D-Ser. Therefore, Y261 is not a key residue in the cleavage of D-Ser. With H80Q, loss of activity was higher with respect to D-Cys (50%) when compared to βCDA (25%). These observations suggest that His80 and Tyr261 may not be directly involved in the degradation of D-Cys and βCDA. T288E and T288E/T315L mutants did not show any activity with all the three substrates. However, as discussed later, the loss of activity with these mutants is related to absence of bound PLP.

### Crystals of *St*DCyD soaked with D-Cys and βCDA

**Figure 5:**
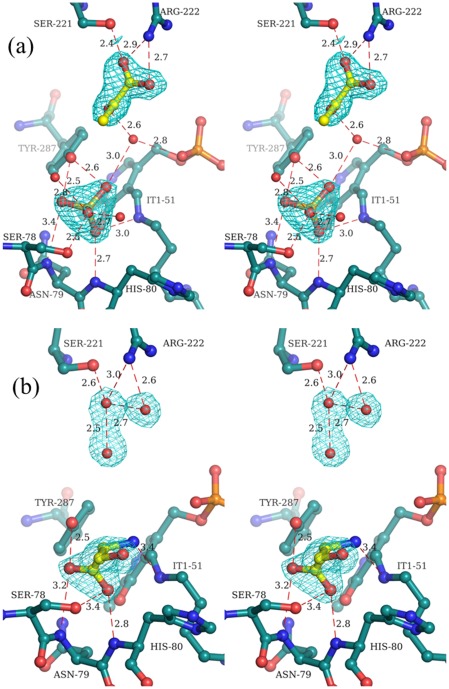
Stereo diagrams illustrating the active sites of *St*DCyD complexes. (a) D-Cys treated *St*DCyD structure shows sulphate at the active site and pyruvate at a site ∼5 Å away. (b) D-Ser-*St*DCyD cocrystallized complex shows D-Ser bound at the active site and water molecules at the second site. Electron density (2Fo-Fc; contoured at 1σ) corresponding to the bound ligands is shown. The distances (Å) of the ligand atoms from active site residues are also shown.

The active sites of *St*DCyD soaked for 30 sec to two min in D-Cys (PDB code: 4D8W) and βCDA (PDB code: 4D92) containing crystallization cocktail show interesting features not previously observed in PLP-dependent enzymes. The additional electron density observed in the complex is consistent with two different binding sites for the substrate. One of these coincides with the canonical active site of fold type II enzymes and the other is 4.0–6.0 Å away from this site. The electron density observed at the active site pocket was not adequate to model the ligand used for soaking the crystals. The density could be modeled as a sulphate and two water molecules. The internal aldimine structure, however, appears to be undisturbed (Fig. 5A). The density at the other site 4.0–6.0 Å away from the active site could be modeled either as product of the catalytic reaction, pyruvate (Fig. 5A) or final intermediate of the reaction, aminoacrylate. Examination of the residual electron density after refinement with various occupancies for pyruvate and the aminoacrylate suggested that it is more likely to represent a pyruvate. The modeled pyruvate is held by hydrogen bonding with Arg222 and Ser221 (Fig. 5A).

Analysis of the *St*DCyD structure using CAVER 2.1 [24] and HotSpotWizard 1.7 [25] programs revealed a channel that might control access of ligands to the active site. The pyruvate binding site occurs in this channel. It has been reported that *Ec*DCyD is inhibited by excess substrate [7]. Thermal melting studies suggest that the T_m_ of *St*DCyD increases from 80° to 85°C in the presence of 10 mM pyruvate (data not shown). Thus, this inhibition might be due to binding of the substrate or product as observed in the structure.

### D-Ser and L-Ser bound structures of *St*DCyD

*St*DCyD does not degrade L-Ser. D-Ser, although analogous to D-Cys, is a poor substrate. Structures of *St*DCyD-D-Ser (PDB code: 4D97) and *St*DCyD-L-Ser (PDB code: 4D99) complexes might provide additional information on the interactions of the substrate with the enzyme. Hence, the structures of *St*DCyD cocrystallized with D- and L-Ser were determined. In these structures, electron density was observed near the active site into which the respective ligand could be fitted. However, the internal aldimine was not disrupted (Fig. 5B and Fig. S1). Presence of non-covalently attached D- and L-Ser at the active site of the structures of these complexes suggests that the formation of external aldimine might be the slowest step in the catalytic reaction for these two substrates. A similar observation was made in the case of *E. coli* D-serine dehydratase [26]. Comparison of these two liganded forms reveals that the enantiomers are oriented with their Cα protons pointing in nearly opposite directions. The proton at the Cα of D-Ser is oriented towards the hydroxyl of Tyr287. The Cα proton in L-Ser is in such a direction that it might approach the ε-amino group of Lys51 in the external aldimine form. Therefore, it is reasonable to suggest that the catalytic reaction with D-Ser is initiated by the abstraction of the Cα proton by Tyr287. This is consistent with the observation that Y287F is completely inactive (Fig. 4). *Ph*AHP has the ability to abstract the Cα-proton from both L and D forms of serine [14]. Comparison of the active site residues of *St*DCyD with those of *Ph*AHP reveals that they differ in only one amino acid. Gln77 of *St*DCyD is replaced by His80 in *Ph*AHP. His80 has been implicated in domain closure in *Ph*AHP although no specific role in the degradation of ACC has been suggested. Q77H mutant of *St*DCyD was less active with respect to all the substrates when compared to the native enzyme (Fig. 4C). As noted earlier, Gln77 in *St*DCyD and His80 in *Ph*AHP are in completely different orientations. Modeling His80 of *Ph*AHP in the orientation similar to that of Gln77 in *St*DCyD leads to unacceptable steric clashes with His316. This residue of *Ph*AHP has been replaced by a smaller residue Ala323 in *St*DCyD and hence no steric clashes are observed between Gln77 and Ala323. The inactivity of Q77H mutant of *St*DCyD with respect to D-Ser may be a result of these substitutions. Therefore, the double mutant Q77H/A323H of *St*DCyD may be active with D-Ser. This needs further exploration. Crystal structures of these mutants and their complexes might provide further insights on the roles of Gln77 of *St*DCyD and the equivalent His80 of *Ph*AHP.

### *St*DCyD co-crystallized with ACC

**Figure 6:**
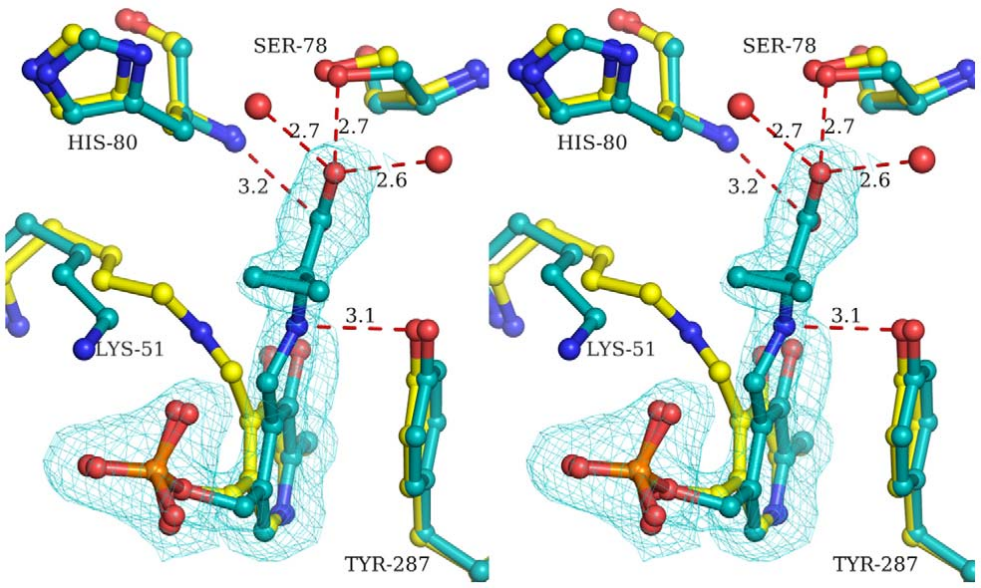
Stereo diagram illustrating the active site of *St*DCyD-ACC complex. Electron density (2Fo-Fc; 1σ) corresponding to the ACC-bound PLP (green) is shown. ACC is bound as an external aldimine. The active site of native *St*DCyD (yellow) is also shown for comparison. PLP is in the internal aldimine form in native *St*DCyD.

Unlike *Ps*ACCD and *Hs*ACCD, *St*DCyD was found to be inactive towards ACC as a substrate. Also, ACC is not a substrate for *Ph*AHP, Y295F mutant of *Hs*ACCD (PDB ID: 1J0E) and K51T mutant of *Hs*ACCD (PDB ID: 1J0D). However, it has been shown that ACC binds to these inactive forms of the enzymes as an external aldimine [14], [15]. Likewise, the structure of *St*DCyD cocrystallized with ACC (PDB code: 4D96) revealed that the ligand binds as an external aldimine (Fig. 6). In this complex, the pyridine ring of PLP is rotated with respect to the internal aldimine form by about 15° and the free ε-amino group of the active site lysine (Lys51) makes a hydrogen bond with the phosphate of PLP. ACC is involved in several non-covalent interactions with the residues lining the substrate binding pocket. The N atom of ACC is hydrogen bonded to the side chain hydroxyl group of Tyr287. Carboxyl group of ACC has hydrogen bonding interactions with main chain atoms of His80 and Asn79, and the side chain hydroxyl of Ser78. It has additional hydrogen bonding interactions with two water molecules present in the active site. Similar binding mode has been observed in *Ph*AHP and K51T mutant of *Hs*ACCD.

The pyridine ring in ACCDs is thought to be a better electron sink due to hydrogen bonding of the pyridine N with a carboxylate group (Glu296 in *Hs*ACCD), which facilitates removal of a proton from the methyl group of the cyclopropane ring of ACC. Indeed, Todorovic and Glick were able to convert tomato DCyD into an ACC deaminase and *Pseudomonas* PW4 ACC deaminase to DCyD by appropriate Glu-Ser mutations [23]. Consistent with these observations, *St*DCyD, which has a threonine (Thr288) at a position equivalent to that of Glu296 of *Hs*ACCD, cannot catalyze the degradation of ACC. In order to examine the importance of hydrogen bonding between pyridine N and a carboxylate, mutants T288E and T315L/T288E of *St*DCyD were constructed and their activities were estimated. Intriguingly, these mutants were unable to bind PLP and therefore were inactive. The side chain carboxyl of Glu296 in *Hs*ACCD is held by hydrogen bonding interactions with Tyr320 hydroxyl and N1 of PLP. Such a hydrogen bonding interaction is not possible with the mutant *St*DCyD due to substitution of Tyr320 of *Hs*ACCD with Phe312 in *St*DCyD. Also, when Thr288 is mutated to glutamate in *St*DCyD and modeled as in *Hs*ACCD, an unacceptable short contact is observed between the mutated residue and N1 of PLP. Thus, T288E mutation might hinder PLP-binding. These observations reflect the subtlety of active site geometry of PLP-dependent enzymes and suggest that concerted mutations are needed to achieve functionally viable active sites endowed with desired specificity.

### Interaction of *St*DCyD with D-cycloserine (DCS) and L-cycloserine (LCS)

**Figure 7:**
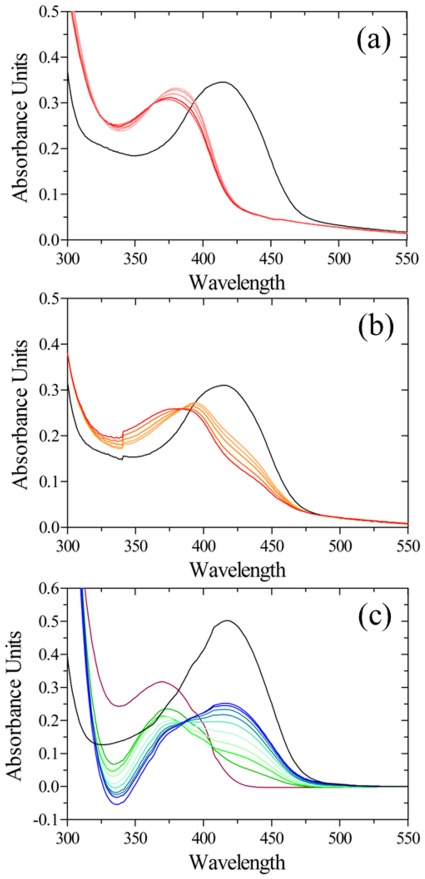
Spectroscopic observations on the interaction of *St*DCyD with cycloserines. Spectral changes at different time points after the addition of the ligand are shown. (a) and (b) correspond to DCS and LCS, respectively. A spectrum of protein before addition of any ligand is shown in black. Orange to bright red represent the spectra at increasing time intervals (1, 2, 5, 10 and 15 min) after the addition of DCS/LCS. The reactions are nearly complete at the end of the first min and probably lead to the formation of oximes characterized by absorption maximum at ∼378 nm. The result of addition of 40 mM pyruvate after allowing the enzyme to react with DCS for 15 min is shown in (c). Purple line represents the spectra with DCS before addition of pyruvate. Green to blue represent spectra at increasing time points (1, 20, 45, 70, 95, 120 and 195 min) after addition of pyruvate. Re-emergence of the 420 nm peak after 4 h is observed.

Examination of the absorption spectrum of *St*DCyD after the addition of 10 mM D-cycloserine (DCS)/L-cycloserine (LCS) revealed a peak at 375 nm that appeared soon after the addition of the ligands. This peak was observed to undergo a slow red shift overnight and stabilize at ∼365 nm (Fig. 7A and 7B). These spectral changes might be due to the formation of an oxime intermediate as proposed in serine palmitoyl transferase [27]. The oxime intermediate could undergo degradation to β-aminoxypyruvate leading to the formation of PMP at the active site. The absorption maximum of PMP is close to 330 nm and not 365 nm as observed in the present case. This might be due to the presence of a mixture of internal aldimine, external aldimine, oxime intermediate forms and PMP. Formation of PMP at the active site might be confirmed by examining the second step of the amino transferase reaction in which a ketoacid is converted to an amino acid, concomitantly with the conversion of PMP to PLP and restoring the absorption maximum at ∼417 nm corresponding to the internal aldimine structure. After overnight incubation of *St*DCyD with DCS/LCS, presence of PMP was examined by the addition of 50 mM pyruvate and observing the spectral changes at periodic intervals. The peak at 420 nm reappeared after 4 h with concomitant loss of peak at 365 nm (Fig. 7C). The absorbance at 420 nm was however lower than that before the addition of DCS/LCS. These observations confirm the formation of PMP and suggest that the reaction of *St*DCyD with DCS or LCS might be similar to that observed in serine palmitoyltransferase [27]. The evidence for the formation of PMP was also obtained from the structure of DCS/LCS-*St*DCyD complexes (see below).

### Cycloserine-Enzyme Complex Structures

**Figure 8:**
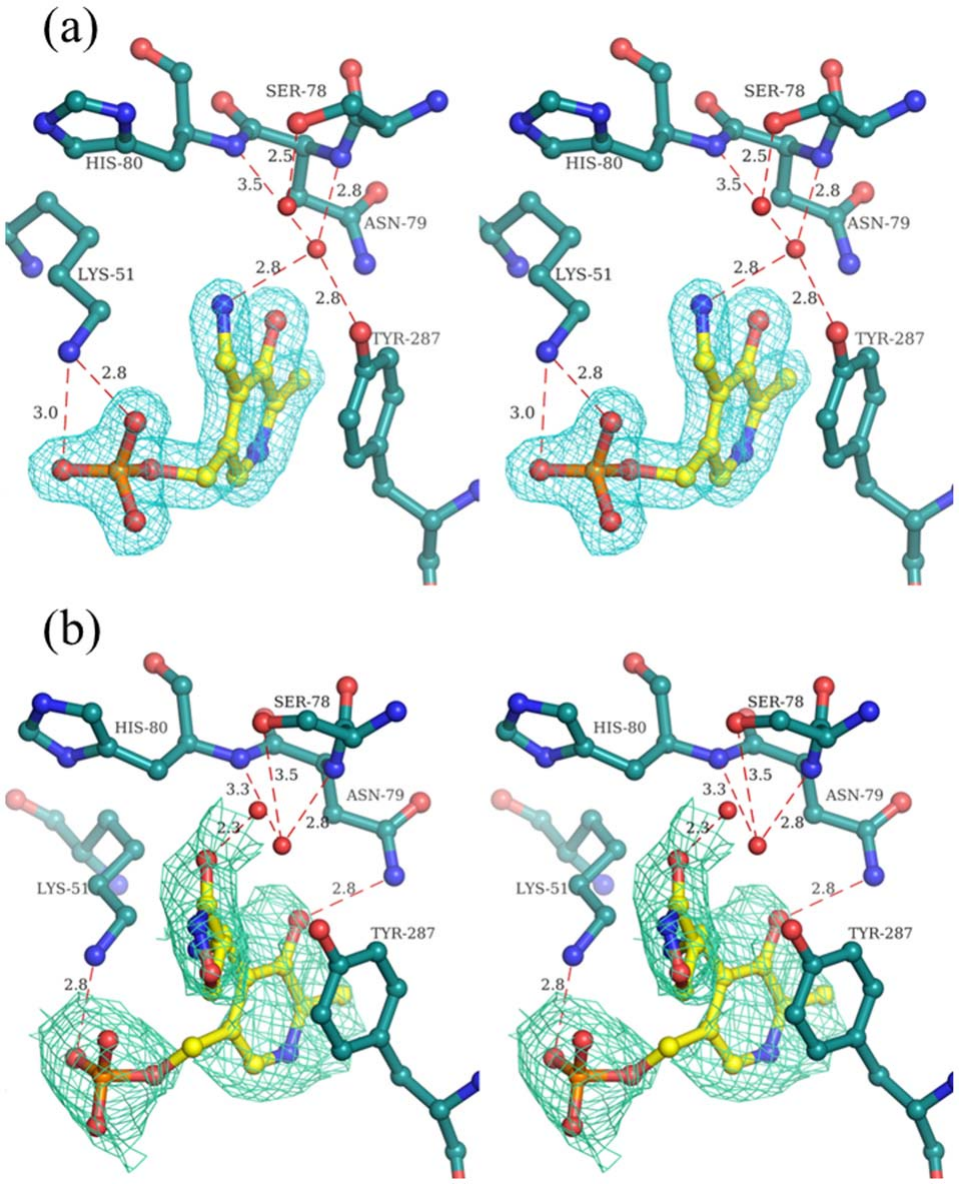
Stereo diagram of the active sites of *St*DCyD cocrystallized or briefly exposed to DCS. (a) PMP bound form obtained by co-crystallization is shown. (b) DCS bound form obtained by brief exposure of preformed *St*DCyD crystals to DCS containing crystallization cocktail is shown. Electron density (2Fo-Fc; 1σ) around PMP and the ligand in *St*DCyD - DCS external aldimine is also shown.

The electron density maps (2Fo-Fc) corresponding to *St*DCyD co-crystallized with DCS/LCS (PDB codes: 4D9B/4D9C) did not show extensive density at the active site into which the ligand or β-aminoxypyruvate could be fitted. The density could be fitted with PLP or PMP and a few water molecules (Fig. 8A). However, the crystals were not yellow and hence the density is likely to correspond to PMP as suggested by spectroscopic observations. PMP is not covalently linked to ε-amino group of Lys51. Side chain conformation of Lys51 is similar to that of the active site lysine in external aldimine form of the enzyme. The density that might correspond to bound sulphate or phosphate observed at the active site of the unliganded enzyme was also absent. In an attempt to trap DCS or LCS at the active site, the crystals of unliganded *St*DCyD were soaked for about two min in mother liquor containing 10 mM DCS (PDB code: 4D9F) or LCS (PDB code: 4D9E), immediately flash frozen in liquid nitrogen and used for diffraction data collection. These crystals diffracted X-rays to 2.5 Å and 2.6 Å, respectively. Electron density observed at the active site was consistent with external aldimines of DCS or LCS (Fig. 8B). Comparison of the unliganded and DCS or LCS bound structures reveal a rotation of the pyridine moiety by about 15° as expected in external aldimine structures. Even here, as in L-Ser and D-Ser complexes, the Cα protons are in opposite orientations. In DCS complex, it points in the direction of phenolate of Tyr287 supporting the fact that phenolate of Tyr287 might be involved in proton abstraction. The active site lysine (Lys51) faces away from the C4′ of the co-factor and is hydrogen bonded to PLP-phosphate. These observations are also consistent with spectroscopic observations (Fig. 7).

### Reaction Mechanism

**Figure 9:**
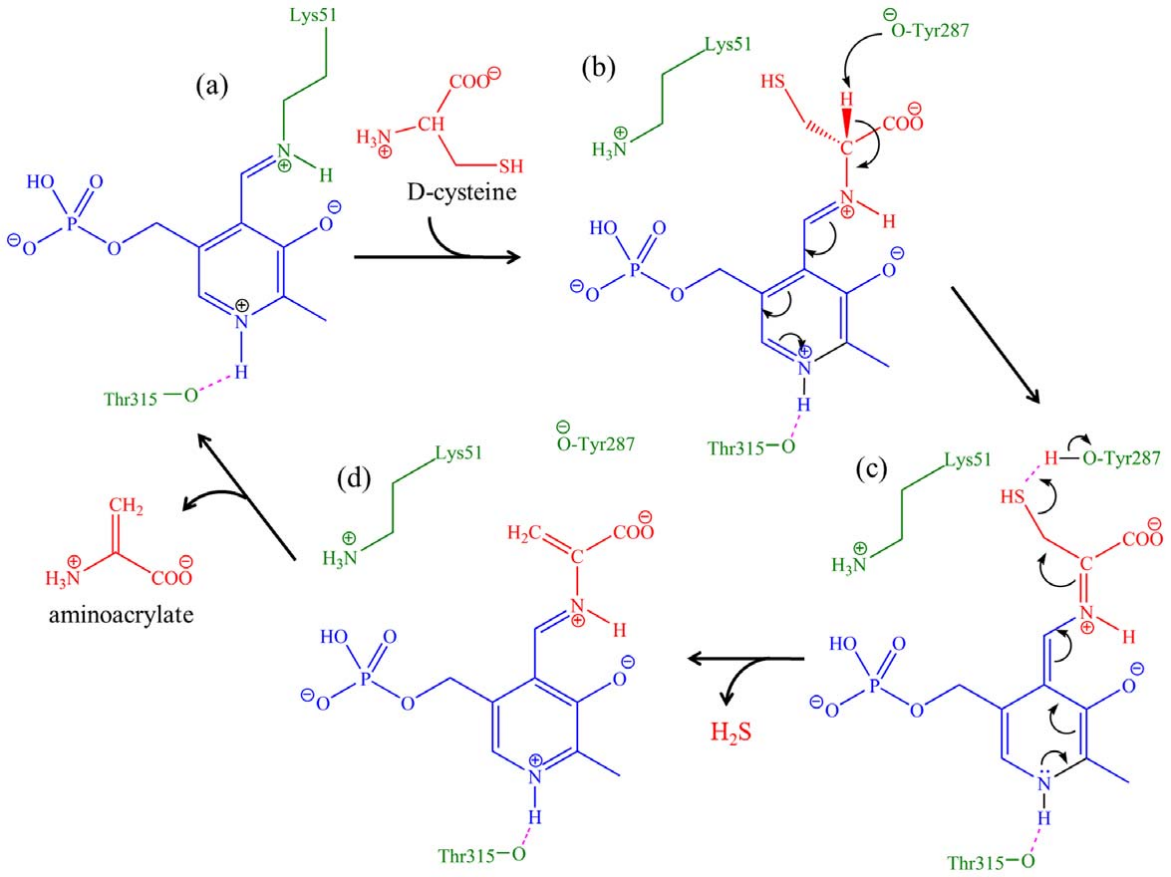
Catalytic mechanism of *St*DCyD. (a) PLP covalently bound to the active site Lys51 as a Schiff base, (b) Imine exchange between the incoming amino group of D-Cys and the internal aldimine leading to the formation of an external aldimine; The Cα proton of the external aldimine faces Tyr287, (c) deprotonation of Cα by the phenolate of Tyr287 facilitating formation of quinonoid complex, (d) protonated Tyr287 releases the -SH group of external aldimine as hydrogen sulfide (H_2_S) leading to its phenolate form and PLP-aminoacrylate complex, (e) a second imine exchange reaction between PLP-aminoacrylate complex and Lys51 regenerating the original PLP-Lys51 Schiff base and releasing aminoacrylate. Aminoacrylate is non-enzymatically cleaved to form pyruvate and ammonia.

A mechanism for the reaction catalyzed by D-serine dehydratase was proposed as early as in 1979 [28]. However due to lack of structural data, the identity of catalytic residues and their role in catalysis could not be unambiguously established. Although the structure of D-serine dehydratase was recently reported [4], it was difficult to discern the catalytic mechanism due to absence of bound PLP. In this context, the structures of *St*DCyD and its complexes presented here for the first time, are important for understanding the catalytic mechanism of PLP-dependent enzymes specific to D-amino acids. Deamination of D-Cys, βCDA and D-Ser by *St*DCyD leads to the release of H_2_S, HCl and H_2_O, respectively. Based on the results presented in this manuscript, the following steps in the reaction catalyzed by *St*DCyD could be envisaged (Fig. 9). The substrate approaches the active site with its carboxyl group pointing towards main chain N atoms of residues Asn79 and His80 and its amino group directed towards the Schiff base of PLP and forms an external aldimine complex (Fig. 9B) releasing Lys51 side chain. Tyr287 may be in its phenolate form due to hydrogen bonding with Tyr261 and is at an appropriate distance for abstraction of Cα proton of the substrate. It has been suggested that the catalytic Lys itself is involved in proton abstraction in L-amino acid deaminases (rat liver L-serine deaminase [9], biodegradative threonine deaminase [10]). A similar role for the active site Lys is unlikely with *St*DCyD as Lys51 is not at an appropriate distance from the Cα of the modeled substrate. Further support for this is provided by the structure of the *St*DCyD-ACC external aldimine, where the ε-amino group of Lys51 and Cα atom of the ligand are at a large distance (5.01 Å). In contrast, in D-serine deaminase from *Salmonella typhimurium* [4] and Serine racemase [29], [30], deprotonation is suggested to be carried out by a Ser or Thr. The presence of non-covalently linked D-Ser and L-Ser at the active site in the D-Ser and L-Ser complexes of *St*DCyD suggests that formation of the external aldimine by transimination may be the slowest step of the catalytic cycle. The deprotonation step leads to the formation of a quinonoid intermediate (Fig. 9C). Further steps in the reaction might be similar to the mechanisms proposed for D-serine dehydratase [28]. The hydroxyl of Tyr287 may donate its proton to the -SH moiety leading to the release of hydrogen sulfide (H_2_S) and formation of PLP-aminoacrylate complex (Fig. 9D). The ε-amino group of Lys51 may then attack the C4′ atom of the pyridine group, regenerating the original lysine-PLP Schiff base and releasing aminoacrylate. Further, hydrolysis of aminoacrylate acid to pyruvate and ammonium ion might occur non-enzymatically [31].

### Conclusions

The structures of *St*DCyD and its complexes presented in this manuscript have provided significant insights on enzyme specificity and differences from other closely related fold type II PLP-dependent enzymes such as ACCDs. *St*DCyD is specific for D-Cys and βCDA, and shows no activity with ACC, although it shares significant sequence identity with ACCDs. The crystals of *St*DCyD obtained in the presence of D-Cys and βCDA show the product pyruvate at a site ∼5 Å away from the substrate binding pocket. Although not a substrate, ACC forms an external aldimine complex. D- and L-Ser bind non-covalently at the active of *St*DCyD without forming external aldimines. Structures of complexes with cycloserines along with the spectroscopic observations suggest that *St*DCyD might cleave D, L-cycloserines also resulting in the formation of PMP at the active site. Based on sequence and structural comparison with ACCDs, a number of mutants were constructed and their catalytic properties were examined. The results suggest that proton abstraction in the first step of catalysis is likely to be carried out by the phenolate of Tyr287. Ser78 and Gln77 appear to be the key determinants of higher specificity towards D-Cys when compared to βCDA. Mutants with amino acid substitutions constructed to modify the active site for ACCD and serine dehydratase activities were not successful reflecting the subtlety of the active site geometry of PLP-dependent enzymes. However, the structure suggests additional mutations that could be carried out for engineering related functions.

## Materials and Methods

### Cloning, over-expression and purification

The *St*DCyD gene was amplified by the polymerase chain reaction (PCR) from *Salmonella typhimurium* genomic DNA with primers designed to introduce *Nhe*I and *Nde*I sites before the initial ATG codon and *Bam*HI and *Xho*I sites at the end of the polypeptide chain. Between *Bam*HI and *Xho*I sites, a stop codon was introduced. The PCR product was digested with *Nhe*I and *Bam*HI and then inserted between the *Nhe*I and *Bam*HI sites of pRSET C vector. The plasmid insert was sequenced to confirm the clone. The clone contained 14 amino acids at the N-terminus (MRGSHHHHHHGMAS) including a hexa-histidine tag that helped in the purification of the expressed protein. The pRSET C-*St*DCyD plasmid was transformed into *E. coli* BL21 (DE3) Rosetta cells. The cells were grown at 37°C in LB medium containing 100 µg ml^-1^ ampicillin till OD at 600 nm reached 0.5 and expression of the cloned gene was induced by the addition of 0.3 mM IPTG. Cells were then allowed to grow at 30°C for another 6 h period. Later, the cells were pelleted by centrifugation at 4817 g for about 10 min and the pellet obtained was resuspended in buffer A containing 50 mM Tris pH 8.0, 400 mM NaCl and 30% glycerol. After sonication and centrifugation, 1 ml of Ni-NTA beads were added to 30 ml of supernatant containing the soluble fraction of the expressed protein, kept for end-to-end rotation for three hours and loaded onto a column. The proteins non-specifically bound to the column were washed using buffer B (50 mM Tris pH 8.0, 200 mM NaCl) followed by wash with buffer B containing 20 mM imidazole and then the protein was eluted using buffer B containing 200 mM imidazole. The eluted protein was concentrated to 1 ml and then loaded onto a size exclusion chromatography column. The protein was eluted with a buffer containing 25 mM Tris pH 8.0 and 50 mM NaCl. The purified protein was concentrated to 10 mg ml^−1^ in centricon tubes and then used for crystallization. Examination of the purified protein on 12% SDS-PAGE showed a single band corresponding to 36 kDa. The molecular weight was confirmed by MALDI-TOF. Analytical gel filtration results showed that the protein is a dimer in solution. Dynamic light scattering experiments showed particles with a radius of gyration of 34 Å and an estimated molecular mass of 72 kDa. These values are also consistent with a dimeric form of the enzyme.

### Biochemical assay and interaction with inhibitors

The activity of the enzyme was measured by a coupled enzyme spectrophotometric method. The enzyme synthesizes pyruvate from D-Cys. Pyruvate is utilized by lactate dehydrogenase with concomitant oxidation of NADH (absorption maximum 340 nm) to NAD^+^. The assay mixture of 1 ml contained 1 µg of the enzyme in Tris buffer pH 8.0, varying concentration of either D-Cys or βCDA, 3.43 units of lactate dehydrogenase, 200 µM NADH. The reaction was initiated by the addition of substrate. The rate of NADH consumption was monitored by recoding the absorbance at 340 nm for five minutes. The substrate concentration dependence of absorbance followed a typical Michaelis-Menton curve. K_m_ and V_max_ of the enzyme for its physiological substrate (D-Cys) and for βCDA were determined. Activity with ACC, D-Ala and L-Ser were also tested. The enzyme was not found to be active with these ligands.

Binding of ligands (D-Cys, βCDA, ACC, D-Ser, L-Ser, DCS and LCS) was monitored by recording the changes in the absorbance spectrum of the enzyme upon addition of ligands using a JASCO UV-visible spectrophotometer. Spectral scans of *St*DCyD with ligands were obtained in 50 mM Tris pH 8.0 buffer containing 100 mM NaCl over a total period of 10 min. The spectral scans (between 300 to 550 nm), were recorded at intervals of 1, 5 and 10 min after the addition of the ligand.

### Crystallization and data collection

Yellow coloured crystals of *St*DCyD were obtained in two distinct forms: form I and form II. The yellow colour of the crystals, as in other PLP dependent enzymes, is due to the internal aldimine form of the enzyme where PLP is covalently bound to an active site lysine through a Schiff base. Crystals of ACC, D-Ser and L-Ser complexes of *St*DCyD were obtained by co-crystallization. Preformed crystals of unliganded *St*DCyD were soaked in D-Cys, βCDA, DCS and LCS for about two min to obtain crystals of the corresponding complexes. The crystals obtained in the presence of D-Cys, βCDA, ACC, D-Ser and L-Ser were also yellow in colour, suggesting the presence of PLP, either as an internal or as an external aldimine. Crystals of *St*DCyD obtained in the presence of DCS and LCS were colourless. Also, preformed yellow crystals of the native enzyme became colourless when soaked in solutions containing either DCS or LCS.

Form I crystals of *St*DCyD were grown by the hanging drop vapor diffusion method at room temperature (25°C). The protein sample (2.5 µl of 8 mg ml^−1^ in 50 mM HEPES pH 7.5) was mixed with 3 µl of the reservoir solution containing 1.5 M ammonium sulphate, 15% (v/v) ethylene glycol and 0.1 M HEPES pH 8.6. To this mixture, 0.5 µl of 2% benzamidine hydrochloride was added. Crystals grew to their full size (100×15×10 µm) in 1 to 2 days. Single crystals were harvested, cryoprotected in 50 mM HEPES (pH 8.6), 1.5 M ammonium sulphate, 20% (v/v) ethylene glycol, 10–12% (w/v) sucrose, and then flash frozen in nitrogen gas at 100 K. Co-crystallization and soaking experiments with various ligands were carried out with form I crystals. Form II crystals of *St*DCyD were obtained under the following three conditions: (a) 1.4 M Na/K phosphate pH 8.2, (b) 0.4 M NaCl, 10 mM MgCl_2_, 5 mM CTAB and (c) 0.2 M tri-sodium citrate, 50 mM HEPES pH 7.5, 20% isopropanol. Crystal growth could be optimized only in 1.4 M Na/K phosphate pH 8.2 with or without 20% ethylene glycol. Single crystals were harvested, cryoprotected with 20% ethylene glycol in mother liquor and flash frozen.

X-ray diffraction data on (i) form I crystals soaked with DCS and LCS and (ii) form I crystals grown in the presence of D-Ser and L-Ser were collected at the European Synchrotron Radiation Facility (Grenoble, France) on beam line BM14 (tuned for maximum flux at 0.97 Å with a 100 µm beam aperture) equipped with a CCD detector. In each case, a single crystal was used to collect a total of 720 frames of rotation data with 0.5° oscillation and 10 sec exposure per frame. All data were processed using HKL2000 and SCALEPACK [32]. X-ray diffraction data on (i) unliganded form II crystal, (ii) unliganded form I crystal, (iii) form I crystal grown in the presence of ACC and (iv) form I crystal soaked in D-Cys or βCDA were collected using a Rigaku Ultrax rotating anode X-ray generator and a Mar research image plate detector system. These data were processed using MOSFLM and SCALA of the CCP4 suite [33]. Form I crystals belonged to the space group P2_1_ whereas the form II crystals belonged to the space group C2. Data collection statistics for the two native forms are given in Table 1.

**Table 1:** Data collection and refinement statistics of native StDCyD.

Data set	Native -4mer	Native -8mer
**Crystal parameters**		
Space group	P2_1_	C2
Unit cell parameters		
a, b, c (Å), β(°)	66.48, 165.45, 69.21, 119.01	181.46, 158.14, 181.93, 94.10
**Data collection**		
Resolution range (Å)	60.45-2.21 (2.33-2.21)	90.66-3.30 (3.48-3.30)
R merge^b^	0.069 (0.201)	0.123 (0.425)
R pim^c^	0.044 (0.131)	0.085 (0.297)
Total no. of reflections	209,277 (26,528)	169,632 (23,835)
No. of unique reflections	63,892 (8,500)	69,703 (9,823)
Mean (I)/σ(I)^d^	11.8 (5.1)	6.7 (2.0)
Completeness (%)	98.4 (90.0)	90.7 (87.9)
Mosaicity	1.01	0.74
Multiplicity	3.3 (3.1)	2.4 (2.4)
Wilson B	23.5	60.0
**Refinement**		
R (%)^e^	18.87	21.54
Rfree (%)^f^	24.18	26.93
No. of atoms		
Protein atoms	10,235	19,159
Ligand atoms	117	232
Solvent atoms	450	18
**Model quality**		
RMS deviation from ideal values		
Bond length (Å)	0.006	0.015
Bond angle (deg.)	1.171	1.690
Dihedral angles (deg.)	5.536	6.154
Average B factor (Å^2^)		
Protein atom	33.82	68.14
Ligand atoms	29.74	65.10
Solvent atoms	35.50	22.72
Residues in Ramachandran plot (%)		
Most favoured regions	90.0	87.4
Allowed regions	9.1	10.5
Generously allowed regions	0.8	1.5
Disallowed regions	0.0	0.6

a Values in parentheses refer to the highest resolution shell.

b Rmerge = (Σ_hkl_Σ_i_|I_i_(hkl)−< I(hkl)>|)/Σ_hkl_ΣI_i_(hkl), where I_i_(hkl) is the intensity of the i^th^ measurement of reflection (hkl) and < I(hkl) > is its mean intensity.

c Rpim = (Σ_hkl_[1/N-1]^1/2^Σ_i_|I_i_(hkl) − < I(hkl) >|)/Σ_hkl_ΣI_i_(hkl), where I_i_(hkl) is the intensity of the i^th^ measurement of reflection (hkl), < I(hkl) > is its mean intensity and N is the number of measurements (redundancy).

d I is the integrated intensity and σ(I) is the estimated standard deviation of that intensity.

e Rwork = (Σ_hkl_|Fo−Fc|)/Σ_hkl_Fo where Fo and Fc are the observed and calculated structure factors.

f Rfree is calculated as for Rwork but from a randomly selected subset of the data (5%), which were excluded from the refinement process.

### Structure solution and refinement

The form I crystals belonged to the space group P2_1_ and diffracted to better than 2.2 Å resolution. The asymmetric unit contained two dimers with a solvent content of 46.8% (*V_M_* = 2.31 Å^3^ Da^−1^). The program PHASER of CCP4 suite was used to carry out molecular replacement calculations using the structure *Ph*AHP (1J0B; sequence identity with *St*DCyD 39%) as the phasing model. The native structure was refined to 2.2 Å resolution with R and R-free values of 0.19 and 0.24, respectively (Table 1). The crystals contained four *St*DCyD protomers in the asymmetric unit. Packing of molecules in this crystal form is influenced by the binding of benzamidine molecules included in the crystallization cocktail. The asymmetric unit has three benzamidine molecules. The form II crystals belonged to the space group C2 and diffracted to a maximum resolution of 3.3 Å. These crystals contained four dimers of *St*DCyD in the asymmetric unit (Fig. 4B) with a solvent content of 72.8% (*V_M_* = 4.52 Å^3^ Da^−1^). The structure of form II crystals was obtained using the dimeric structure of form I as the phasing model. The model was refined to R and R-free of 0.21 and 0.26, respectively (Table 1).

The liganded structures of *St*DCyD with D-Cys, βCDA, ACC, D-Ser, L-Ser, DCS and LCS were determined using the dimeric structure of unliganded *St*DCyD (form I) as the phasing model. Models fitted into the resulting 2Fo-Fc map could be refined with reasonable stereochemical parameters. Data collection and structure refinement statistics of all the ligand-complex structures of *St*DCyD are given in supplementary tables, Table S1, Table S2 and Table S3.

### Structure analysis

The geometry of the final models was checked using PROCHECK and MOLPROBITY. Structural superposition of various liganded and native forms was achieved using the programs ALIGN [34] and SSM superpose [35] feature of COOT [36]. ALIGN was also used for comparing the position and conformation of the active site residues in these structures. Average B-factors for protein atoms, water molecules, and ligands were calculated using BAVERAGE of CCP4 suite. Interactions were evaluated using CONTACT program of the CCP4 suite. Identification of the interface residues and their interactions, surface area and examination of the plausible physiological dimeric state were performed using the PISA server/program. All the figures were made using PyMOL [37].

### Site directed mutagenesis

Single site mutants of Ser78, Tyr287, Thr315, Tyr261, His80 and Gln77 were constructed by site directed mutagenesis utilizing single primer extension method [38]. *St*DCyD cloned in pRSET C vector was used as the template for generation of mutants. The double mutant T315L/T288E was constructed from the T315L clone. All mutants were confirmed by sequencing.

### Catalytic properties of mutants

Pyruvate released from D-Cys, βCDA and D-Ser by the enzymatic action of *St*DCyD was estimated using the 2,4-dinitrophenylhydrazine (DNPH) method [39]. The reaction mixture for the assay with D-Cys consisted of 50 mM Tris buffer (pH 8.0), 20 µM PLP and 2 mM D-Cys and 1 µg of *St*DCyD in a final volume of 50 µl. The reaction was started by the addition of D-Cys and carried out at 22°C for 5 min. 50 µl of 0.1% 2,4-DNPH in 2 M HCl was added to stop the reaction and the mixture was incubated at 37°C for two min, followed by the addition of 150 µl of 1 M NaOH. After five min incubation at room temperature, OD_540_ of the hydrazone product formed was measured. The resulting absorbance units were corrected for enzyme-blank. Similar procedure was followed for assays with βCDA and D-Ser. 5 mM βCDA and 50 mM D-Ser were incubated at 22°C and 37°C for 10 min and 30 min respectively.

## Supporting Information

Figure S1 **Stereo diagram of the active site of** ***St*** **DCyD co-crystallized with L-Ser.** Electron density (2Fo–Fc; 1σ, green) corresponding to L-Ser (yellow; ball and stick) and two water molecules (red spheres) near Ser221 and Arg222 is shown. Distances shown are in Å.
(TIF)

Table S1 **Data collection and refinement statistics of** ***St*** **DCyD bound to β-chloro-D-alanine (βCDA), D-Cys and amino carboxy cyclopropane (ACC).**
(DOC)

Table S2 **Data collection and refinement statistics of** ***St*** **DCyD bound to D-Ser and L-Ser.**
(DOC)

Table S3 **Data collection and refinement statistics of** ***St*** **DCyD bound to inhibitors D-cycloserine and L-cycloserine (DCS and LCS were collected at ESRF.** These crystals were obtained by cocrystallization with respective ligands. dcs1 and lcs1 were collected at homesource. These complexes were obtained by soaking experiments).
(DOC)
