# Supplementary figures and images for: Structural and Mutational Studies on Substrate Specificity and Catalysis of Salmonella typhimurium D-Cysteine Desulfhydrase
Source: PLoS One. 2012 May 4;7(5):e36267. doi: 10.1371/journal.pone.0036267 (PMC3344862; doi:10.1371/journal.pone.0036267)

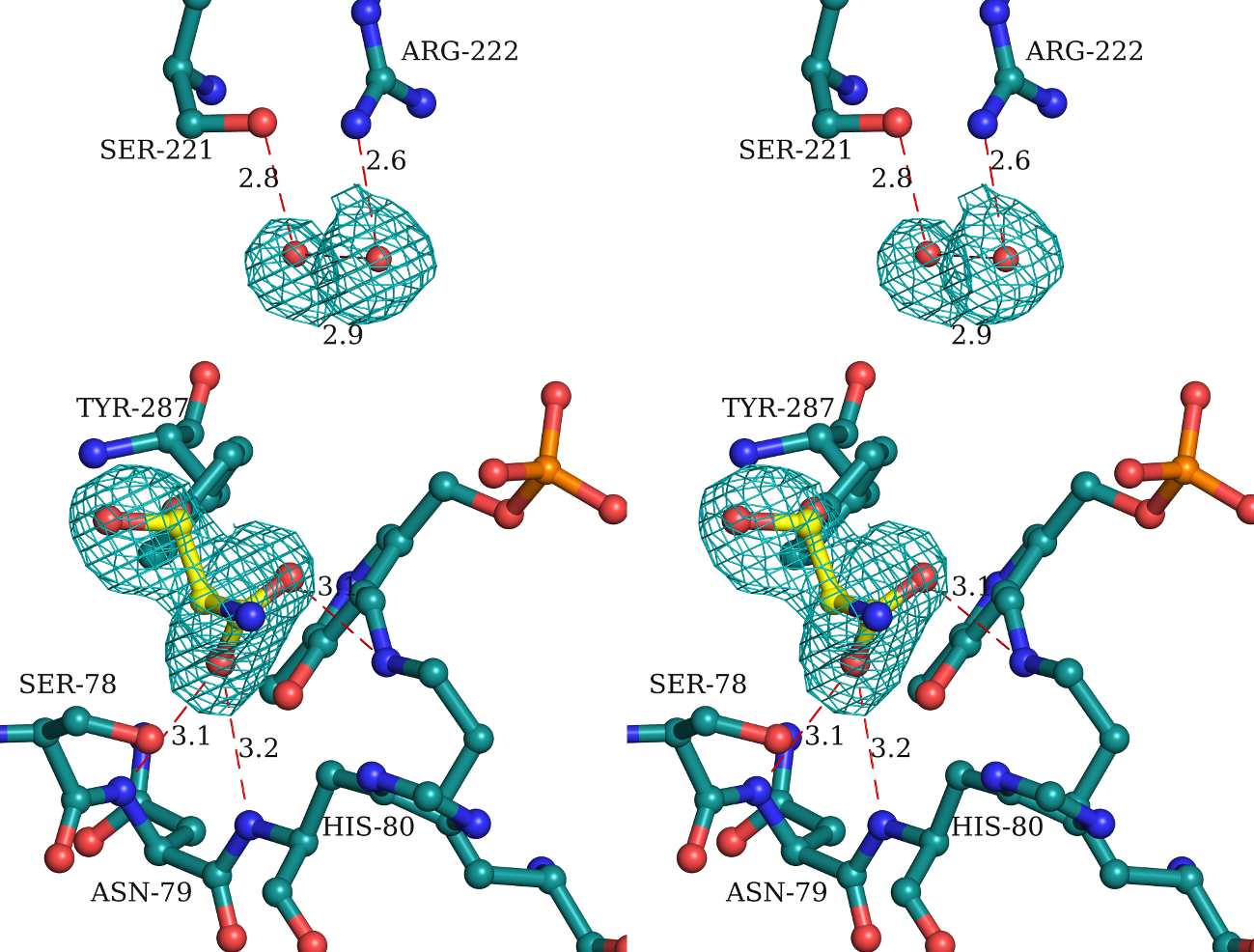

Supplement: Figure S1 — Stereo diagram of the active site of St DCyD co-crystallized with L-Ser. Electron density (2Fo–Fc; 1σ, green) corresponding to L-Ser (yellow; ball and stick) and two water molecules (red spheres) near Ser221 and Arg222 is shown. Distances shown are in Å. (TIF) [file pone.0036267.s001.tif]
