# Supplementary material for: Structural and Mutational Studies on Substrate Specificity and Catalysis of Salmonella typhimurium D-Cysteine Desulfhydrase
Source: PLoS One. 2012 May 4;7(5):e36267. doi: 10.1371/journal.pone.0036267 (PMC3344862; doi:10.1371/journal.pone.0036267)
Supplement: Table S1 — Data collection and refinement statistics of St DCyD bound to β-chloro-D-alanine (βCDA), D-Cys and amino carboxy cyclopropane (ACC). (DOC) [file pone.0036267.s002.doc]

Table S1 : Data collection and refinement statistics of *St*DCyD bound to β-chloro-D-alanine (βCDA), D-Cys and amino carboxy cyclopropane (ACC).

| **Data set**^a^ | **3-Chloro D-Alanine** | **D-cysteine** | **Amino carboxy cyclopropane** |
| --- | --- | --- | --- |
| **Crystal parameters** |  |  |  |
| Space group | P21 | P21 | P21 |
| Unit cell parameters |  |  |  |
| a, b, c (Å), β(^o^) | 66.34, 165.46, 67.96, 118.50 | 66.29, 165.19, 68.06, 118.71 | 66.59, 165.35, 68.92, 119.01 |
| **Data collection** |  |  |  |
| Resolution range (Å) | 59.72- 2.21 (2.33-2.21) | 48.38 – 2.00 (2.11 – 2.00) | 55.12-2.07 (2.18-2.07) |
| R merge^b^ | 0.069 (0.292) | 0.066 (0.341) | 0.077 (0.219) |
| R pim^c^ | 0.042 (0.177) | 0.040 (0.212) | 0.047 (0.135) |
| Total no. of reflections | 206,155 (23,082) | 316,676 (38,677) | 255,134 (31,430) |
| No. of unique reflections | 58,457 (6,842) | 85,215 (11,081) | 70,270 (9,020) |
| Mean (I)/σ(I)^d^ | 14.4 (4.2) | 15.1 (3.6) | 11.4 (4.8) |
| Completeness (%) | 91.6 (73.7) | 98..2 (87.9) | 88.6 (78.2) |
| Mosaicity | 0.49 | 0.57 | 0.86 |
| Multiplicity | 3.5 (3.4) | 3.7 (3.5) | 3.6 (3.5) |
| Wilson B | 24.6 | 21.0 | 22.9 |
| **Refinement** |  |  |  |
| R (%)^e^ | 17.59 | 18.31 | 21.49 |
| Rfree (%)^f^ | 22.22 | 21.72 | 26.40 |
| No. of atoms |  |  |  |
| Protein atoms | 10,363 | 10,615 | 10,469 |
| Ligand atoms | 167 | 162 | 125 |
| Solvent atoms | 586 | 748 | 639 |
| **Model quality** |  |  |  |
| RMS deviation from ideal values |  |  |  |
| Bond length (Å) | 0.006 | 0.005 | 0.023 |
| Bond angle (^o^) | 1.125 | 1.097 | 2.056 |
| Dihedral angles (^o^) | 5.452 | 5.426 | 6.734 |
| Average B factor (Å^2^) |  |  |  |
| Protein atom | 22.43 | 22.48 | 21.93 |
| Ligand | 15.11 | 19.36 | 18.54 |
| Water | 26.04 | 27.50 | 24.87 |
| Residues in Ramachandran plot (%) |  |  |  |
| Most favoured regions | 90.6 | 90.9 | 89.4 |
| Allowed regions | 9.0 | 8.7 | 9.6 |
| Generously allowed regions | 0.5 | 0.4 | 1.0 |
| Disallowed regions | 0 | 0 | 0 |

^a^Values in parentheses refer to the highest resolution shell

^b^Rmerge= (Σ_hkl_Σ_i_|I_i_(hkl) - < I(hkl)>|)/Σ_hkl_ΣI_i_(hkl), where I_i_(hkl) is the intensity of the i^th^ measurement of reflection (hkl) and < I(hkl) > is its mean intensity.

^c^Rpim = (Σ_hkl_[1/N-1]^1/2^Σ_i_|I_i_(hkl) - < I(hkl) >|) / Σ_hkl_ΣI_i_(hkl), where I_i_(hkl) is the intensity of the i^th^ measurement of reflection (hkl), < I(hkl) > is its mean intensity and N is the number of measurements (redundancy).

^d^I is the integrated intensity and σ(I) is the estimated standard deviation of that intensity.

^e^Rwork = (Σ_hkl_|Fo-Fc|)/Σ_hkl_Fo where Fo and Fc are the observed and calculated structure factors.

^f^Rfree is calculated as for Rwork but from a randomly selected subset of the data (5%), which were excluded from the refinement process.
