# Supplementary material for: Structural and Mutational Studies on Substrate Specificity and Catalysis of Salmonella typhimurium D-Cysteine Desulfhydrase
Source: PLoS One. 2012 May 4;7(5):e36267. doi: 10.1371/journal.pone.0036267 (PMC3344862; doi:10.1371/journal.pone.0036267)
Supplement: Table S2 — Data collection and refinement statistics of St DCyD bound to D-Ser and L-Ser. (DOC) [file pone.0036267.s003.doc]

Table S2 : Data collection and refinement statistics of *St*DCyD bound to D-Ser and L-Ser

| **Data set**^a^ | **D-Ser** | **L-Ser** |
| --- | --- | --- |
| **Crystal parameters** |  |  |
| Space group | P21 | P21 |
| Unit cell parameters |  |  |
| a, b, c (Å), β(^o^) | 66.45, 165.29, 68.7, 118.71 | 66.56, 165.49, 68.80, 119.34 |
| **Data collection** |  |  |
| Resolution range (Å) | 50.00- 1.77 (1.80-1.77) | 50.00-2.00 (2.03-2.00) |
| R merge^b^ | 0.089 (0.678) | 0.060 (0.196) |
| Total no. of reflections | 918,910 | 560,361 |
| No. of unique reflections | 124,064 (5,888) | 78,475 (4,026) |
| Mean (I)/σ(I)^d^ | 28.14 (1.83) | 44.72 (8.39) |
| Completeness (%) | 99.2 (94.3) | 91.1 (94.8) |
| Multiplicity | 7.4 (5.6) | 7.1 (6.6) |
| **Refinement** |  |  |
| R (%)^e^ | 21.56 | 21.18 |
| Rfree (%)^f^ | 24.77 | 25.89 |
| No. of atoms |  |  |
| Protein atoms | 10,536 | 10,364 |
| Ligand atoms | 151 | 152 |
| Solvent atoms | 690 | 594 |
| **Model quality** |  |  |
| RMS deviation from ideal values |  |  |
| Bond length (Å) | 0.005 | 0.006 |
| Bond angle (^o^) | 1.096 | 1.152 |
| Dihedral angles (^o^) | 5.334 | 5.637 |
| Average B factor (Å^2^) |  |  |
| Protein atom | 25.91 | 32.2 |
| Ligand | 23.97 | 32.03 |
| Water | 31.37 | 35.56 |
| Residues in Ramachandran plot (%) |  |  |
| Most favoured regions | 90.7 | 89.7 |
| Allowed regions | 8.6 | 9.5 |
| Generously allowed regions | 0.5 | 0.6 |
| Disallowed regions | 0.2 | 0.2 |

^a^Values in parentheses refer to the highest resolution shell

^b^Rmerge= (Σ_hkl_Σ_i_|I_i_(hkl) - < I(hkl)>|)/Σ_hkl_ΣI_i_(hkl), where I_i_(hkl) is the intensity of the i^th^ measurement of reflection (hkl) and < I(hkl) > is its mean intensity.

^c^Rpim = (Σ_hkl_[1/N-1]^1/2^Σ_i_|I_i_(hkl) - < I(hkl) >|) / Σ_hkl_ΣI_i_(hkl), where I_i_(hkl) is the intensity of the i^th^ measurement of reflection (hkl), < I(hkl) > is its mean intensity and N is the number of measurements (redundancy).

^d^I is the integrated intensity and σ(I) is the estimated standard deviation of that intensity.

^e^Rwork = (Σ_hkl_|Fo-Fc|)/Σ_hkl_Fo where Fo and Fc are the observed and calculated structure factors.

^f^Rfree is calculated as for Rwork but from a randomly selected subset of the data (5%), which were excluded from the refinement process.
