# Supplementary material for: Structural and Mutational Studies on Substrate Specificity and Catalysis of Salmonella typhimurium D-Cysteine Desulfhydrase
Source: PLoS One. 2012 May 4;7(5):e36267. doi: 10.1371/journal.pone.0036267 (PMC3344862; doi:10.1371/journal.pone.0036267)
Supplement: Table S3 — Data collection and refinement statistics of St DCyD bound to inhibitors D-cycloserine and L-cycloserine (DCS and LCS were collected at ESRF. These crystals were obtained by cocrystallization with respective ligands. dcs1 and lcs1 were collected at homesource. These complexes were obtained by soaking experiments). (DOC) [file pone.0036267.s004.doc]

Table S3: Data collection and refinement statistics of *St*DCyD bound to inhibitors D-cycloserine and L-cycloserine (DCS and LCS were collected at ESRF. These crystals were obtained by cocrystallization with respective ligands. dcs1 and lcs1 were collected at homesource. These complexes were obtained by soaking experiments).

| **Data set**^a^ | **DCS** | **LCS** | **dcs1** | **lcs1** |
| --- | --- | --- | --- | --- |
| **Crystal parameters** |  |  |  |  |
| Space group | P21 | P21 | P21 | P21 |
| Unit cell parameters |  |  |  |  |
| a, b, c (Å), β(^o^) | 66.32, 164.93, 68.31, 118.97 | 66.51, 165.50, 68.64, 118.89 | 66.16, 167.51, 68.77, 121.28 | 66.42, 165.84, 68.06, 119.03 |
| **Data collection** |  |  |  |  |
| Resolution range (Å) | 50.00- 1.67  (1.73-1.67) | 50.00-1.96  (1.99-1.96) | 58.78-2.59  (2.73-2.59) | 59.51-2.44  (2.57-2.44) |
| R merge^b^ | 0.053 (0.271) | 0.041 (0.10) | 0.073 (0.161) | 0.103 (0.232) |
| Total no. of reflections | 574,384 | 466,000 | 78,980 (8,395) | 143,152 (12,463) |
| No. of unique reflections | 143,652 (13,251) | 91,865 (4,069) | 32,824 (3,868) | 41,742 (4,058) |
| Mean (I)/σ(I)^d^ | 33.94 (3.68) | 49.53 (14.66) | 9.0 (4.0) | 8.2 (4.2) |
| Completeness (%) | 96.9 (89.9) | 99.4 (89.5) | 83.0 (66.4) | 87.3 (58.7) |
| Multiplicity | 4.0 (3.6) | 5.1(4.5) | 2.4 (2.2) | 3.4 (3.1) |
| **Refinement** |  |  |  |  |
| R (%)^e^ | 17.05 | 17.51 | 23.9 | 22.8 |
| Rfree (%)^f^ | 20.59 | 23.14 | 28.8 | 28.3 |
| No. of atoms |  |  |  |  |
| Protein atoms | 10,773 | 10,545 | 9,876 | 10,065 |
| Ligand atoms | 115 | 91 | 115 | 115 |
| Solvent atoms | 887 | 745 | 175 | 248 |
| **Model quality** |  |  |  |  |
| RMS deviation from ideal values |  |  |  |  |
| Bond length (Å) | 0.028 | 0.026 | 0.013 | 0.017 |
| Bond angle (^o^) | 2.231 | 2.039 | 1.616 | 1.94 |
| Dihedral angles (^o^) | 6.516 | 6.649 | 6.581 | 6.415 |
| Average B factor (Å^2^) |  |  |  |  |
| Protein atom | 22.17 | 23.25 | 29.32 | 22.38 |
| Ligand | 20.48 | 24.05 | 31.63 | 19.86 |
| Water | 29.93 | 29.98 | 18.74 | 21.93 |
| Residues in Ramachandran plot (%) |  |  |  |  |
| Most favoured | 90.5 | 90.6 | 87.7 | 88.9 |
| Allowed | 9.0 | 8.7 | 11 | 9.8 |
| Generously allowed | 0.5 | 1.6 | 1.3 | 1.1 |
| Disallowed | 0.0 | 0.1 | 0.0 | 0.2 |

^a^Values in parentheses refer to the highest resolution shell

^b^Rmerge= (Σ_hkl_Σ_i_|I_i_(hkl) - < I(hkl)>|)/Σ_hkl_ΣI_i_(hkl), where I_i_(hkl) is the intensity of the i^th^ measurement of reflection (hkl) and < I(hkl) > is its mean intensity.

^c^Rpim = (Σ_hkl_[1/N-1]^1/2^Σ_i_|I_i_(hkl) - < I(hkl) >|) / Σ_hkl_ΣI_i_(hkl), where I_i_(hkl) is the intensity of the i^th^ measurement of reflection (hkl), < I(hkl) > is its mean intensity and N is the number of measurements (redundancy).

^d^I is the integrated intensity and σ(I) is the estimated standard deviation of that intensity.

^e^Rwork = (Σ_hkl_|Fo-Fc|)/Σ_hkl_Fo where Fo and Fc are the observed and calculated structure factors.

^f^Rfree is calculated as for Rwork but from a randomly selected subset of the data (5%), which were excluded from the refinement process.
